# The Effectiveness of a Multidisciplinary Electronic Discharge Readiness Tool: Prospective, Single-Center, Pre-Post Study

**DOI:** 10.2196/27568

**Published:** 2021-11-08

**Authors:** Angela Keniston, Lauren McBeth, Jonathan Pell, Kasey Bowden, Anna Metzger, Jamie Nordhagen, Amanda Anthony, John Rice, Marisha Burden

**Affiliations:** 1 Anschutz Medical Campus Division of Hospital Medicine University of Colorado Aurora, CO United States; 2 Colorado School of Public Health University of Colorado Aurora, CO United States; 3 UCHealth Denver, CO United States; 4 Adult and Child Center for Health Outcomes Research and Delivery Science University of Colorado Aurora, CO United States

**Keywords:** discharge planning, health information technology, quasi-experimental design, multidisciplinary, teamwork

## Abstract

**Background:**

In the face of hospital capacity strain, hospitals have developed multifaceted plans to try to improve patient flow. Many of these initiatives have focused on the timing of discharges and on lowering lengths of stay, and they have met with variable success. We deployed a novel tool in the electronic health record to enhance discharge communication.

**Objective:**

The aim of this study is to evaluate the effectiveness of a discharge communication tool.

**Methods:**

This was a prospective, single-center, pre-post study. Hospitalist physicians and advanced practice providers (APPs) used the Discharge Today Tool to update patient discharge readiness every morning and at any time the patient status changed throughout the day. Primary outcomes were tool use, time of day the clinician entered the discharge order, time of day the patient left the hospital, and hospital length of stay. We used linear mixed modeling and generalized linear mixed modeling, with team and discharging provider included in all the models to account for patients cared for by the same team and the same provider.

**Results:**

During the pilot implementation period from March 5, 2019, to July 31, 2019, a total of 4707 patients were discharged (compared with 4558 patients discharged during the preimplementation period). A total of 352 clinical staff had used the tool, and 84.85% (3994/4707) of the patients during the pilot period had a discharge status assigned at least once. In a survey, most respondents reported that the tool was helpful (32/34, 94% of clinical staff) and either saved time or did not add additional time to their workflow (21/24, 88% of providers, and 34/34, 100% of clinical staff). Although improvements were not observed in either unadjusted or adjusted analyses, after including starting morning census per team as an effect modifier, there was a reduction in the time of day the discharge order was entered into the electronic health record by the discharging physician and in the time of day the patient left the hospital (decrease of 2.9 minutes per additional patient, *P=*.07, and 3 minutes per additional patient, *P=*.07, respectively). As an effect modifier, for teams that included an APP, there was a significant reduction in the time of day the patient left the hospital beyond the reduction seen for teams without an APP (decrease of 19.1 minutes per patient, *P=*.04). Finally, in the adjusted analysis, hospital length of stay decreased by an average of 3.7% (*P=*.06).

**Conclusions:**

The Discharge Today tool allows for real time documentation and sharing of discharge status. Our results suggest an overall positive response by care team members and that the tool may be useful for improving discharge time and length of stay if a team is staffed with an APP or in higher-census situations.

## Introduction

Hospitals around the country, in particular tertiary and quaternary referral centers, can face bottlenecks and capacity issues [1-3]. Successful management of capacity and throughput by hospitals allows increased access for patients who need a higher level of care and expertise [1-4]. Delayed discharge of hospitalized patients can impede the flow of patients throughout a hospital [1,3,5-8], resulting in delays in care for patients being admitted [9,10] and adverse events, including medication errors [11,12], infections [13], and increased mortality [13-16]. Delays in discharge are associated with both increased lengths of stay and costs [2,17-19].

The commonly used discharge communication workflows often hinder efficient, timely discharge [20]. Many hospitals document an expected date of discharge at the time of admission, and triaging of work is based on this information documented very early in the patient admission process; however, patient condition changes frequently throughout hospitalization [21]. Clinicians, nurses, care management, pharmacy, and other team members often meet midmorning or in the afternoon each day to discuss discharge needs for hospitalized patients; however, minimal communication occurs before these meetings or in real time; in addition, these meetings do not integrate well into workflows [22]. The lack of communication early in the day, before rounding on patients, delays discharge communication and, ultimately, patient discharge. Earlier discharge, by as little as 1 hour, has been shown to alleviate hospital crowding, reduce access blocking, and improve patient flow [23,24].

Typical workflows rely on processes implemented outside of the electronic health record (EHR), such as meetings, paging, and telephone calls, which are inadequate for efficient discharge communication and frequently interrupt patient care [25,26]. Health information technology solutions most often described in the literature include passive communication tools, such as electronic patient journey boards, hospital capacity dashboards, asynchronous electronic reports, and discharge checklists [4,27-34], or health information technology tools that reside outside of the EHR [35,36]. Even commonly used tools within the EHR, such as messaging or conditional discharge orders, do not provide real time, integrated communications despite being a function of an EHR [37,38].

To address these deficits, we developed a novel EHR tool to facilitate communication in real time between hospitalists and other clinicians and care team members about discharge readiness and barriers to discharge. We evaluated whether the use of this tool was associated with improvements in discharge order time, discharge time, and length of stay. In addition, we evaluated whether this tool worked differently under different conditions, such as high-census days or when an advanced practice provider (APP) was assigned to a patient team. Finally, we evaluated whether the effects of this tool persisted after formal stakeholder engagement efforts waned.

## Methods

### Tool Development

**Figure 1:**
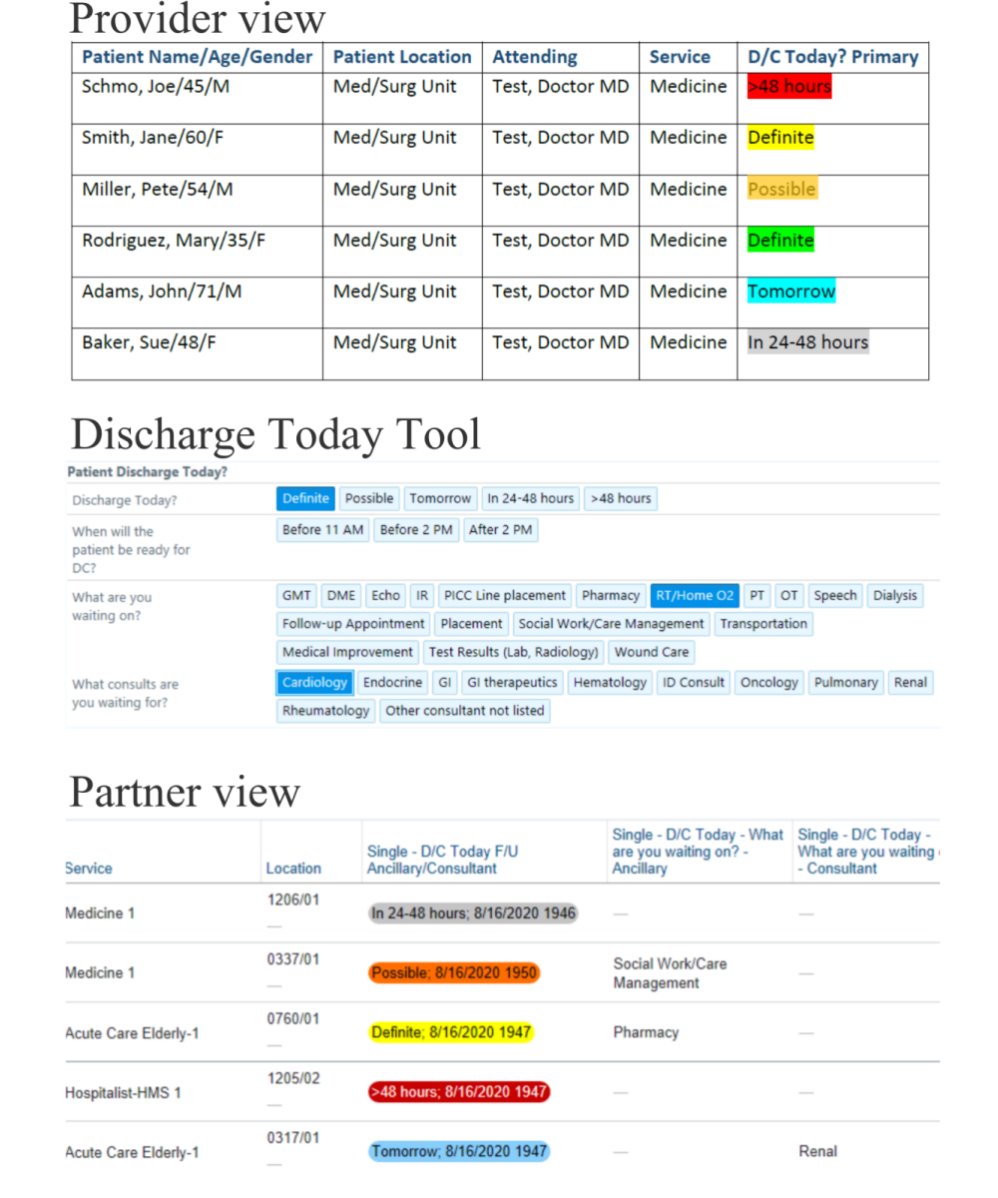
The Discharge Today tool (demo only, no protected health information).

Using multiple user-centered design strategies [39-42], the Discharge Today Tool was iteratively developed from July 1, 2018, to July 31, 2019, and deployed to hospitalists and other clinical staff on March 5, 2019. This tool was designed to integrate with customizable EHR patient worklists used by most clinicians and staff members providing clinical care to hospitalized patients (Figure 1).

In the provider view, hospitalists may access the Discharge Today tool via the D/C Today? Primary column in their EHR patient list. Using this tool, hospitalists may document patient discharge readiness (definite today, possible today, tomorrow, in 24 to 48 hours, or in more than 48 hours) and if the hospitalist is waiting on any final care before the patient can be discharged. Via the partner view, the data collected by the Discharge Today tool is shared with ancillary and consulting clinicians in the Single—D/C Today—What are you waiting on?—Ancillary and the Single—D/C Today—What are you waiting on?—Consultant columns in their EHR patient worklists. The definitions for the discharge readiness statuses are as follows: Definite-very high probability that the patient will be discharged today unless there are unexpected changes during the day. For example, if you have a patient who is clinically ready for discharge but needs home oxygen set up, this patient would be considered a definite discharge, awaiting respiratory therapy. Possible-some probability that the patient could be discharged today. For example, if you have a patient with complex health conditions waiting for subacute nursing facility placement, this patient would be considered a possible discharge, awaiting placement. Tomorrow: very likely that the patient could be discharged tomorrow. In 24-48 hours: the patient is not going home today but will likely be discharged in the next 24 to 48 hours; >48 hours: very unlikely that the patient would be discharged within the next 48 hours.

Hospitalists caring for the patients are able to easily document discharge readiness (definite today, possible today, tomorrow, in 24-48 hours, or in >48 hours) [21] and whether the hospitalist is waiting on any final care before the patient can be discharged. The data collected by the Discharge Today tool are also disseminated via EHR patient worklists, which are EHR-based reports designed to summarize patient care for clinicians using the EHR, and via an automatic paging functionality directly from the EHR. Details can be found in our study describing stakeholder engagement and the user-centered design approaches applied [43].

Addressing communication challenges by improving the efficiency and accuracy of communication may reduce inefficiencies and errors in health care, including during the discharge process. The Discharge Today tool fosters flexibility and agility in communication, including asynchronous communication, feedback loop capabilities, different functionalities according to user role, and allowing for both formal and informal communication.

### Study Design

This study was conducted as a prospective, single-center, quasi-experimental, pre-post study designed to evaluate the effectiveness of the Discharge Today tool. The study was approved by the Colorado Multiple institutional review board as a quality improvement project and funded by a small pilot grant.

### Setting

This study was conducted at the University of Colorado Hospital, a 678-bed tertiary care center with approximately 12,000 medicine discharges per year.

### Inclusion and Exclusion Criteria

Hospitalist physicians and APPs were trained as they started on service and asked to use the Discharge Today tool every day that they were on service with all patients assigned to their team. Clinicians were asked to update patient discharge readiness statuses first thing in the morning and throughout the day as discharge readiness and needs evolved. Patients who were expected to be discharged >48 hours out only needed an update every 3 days as the tool would automatically unpopulate the patient status if unchanged after 3 days to ensure the most accurate and up-to-date information. Clinicians received a small incentive for participation (ie, coffee or other small tokens of gratitude that were funded by the small grant).

Patients were enrolled in this study as part of their regular hospitalization if they presented during the study period. Patients already in the hospital at the start of the pilot implementation period (March 5, 2019, to July 31, 2019) were excluded from the analysis. Patients admitted on or after March 5, 2019, and discharged on or before July 31, 2019, were assigned to the pilot implementation period.

### Data Collection

All patient-level clinical and quality outcomes data queried from the hospital EHR data warehouse were collected as part of their hospitalization process. We queried data from the EHR data warehouse for any patient admitted to the hospital and assigned to a hospital medicine service during the preimplementation period (October 1, 2018, to March 4, 2019), the pilot implementation period (March 5, 2019, to July 31, 2019), and the postimplementation maintenance period (August 1, 2019, to December 31, 2019).

To assess adoption, we documented the number of users who added the tool to their patient worklists within the EHR. To assess both reach and implementation, we queried each time data were entered into the tool by a clinician, including discharge readiness status, when patients assigned a definite discharge status would be ready to be discharged, and what ancillary services or tasks might be needed, such as rehabilitation services, respiratory therapy, pharmacy, social work, care management, medical improvement, or consultant services (Table 1).

**Table 1:** Definitions.

Variable	Definition	Type	Level
Discharge order time	The time of day the physician entered a discharge order for a patient into the electronic health record	Outcome	Patient encounter
Discharge time	The time of day the patient left the hospital after being discharged	Outcome	Patient encounter
Length of stay	The duration, in hours, between admission to the hospital and discharge from the hospital	Outcome	Patient encounter
Team assignment	The team to which the patient was assigned when they were discharged from the hospital	Random effect	Team
Physician	The physician who discharged the patient	Random effect	Physician
Type of patient	Patients admitted for inpatient hospitalization or patients admitted for observation	Confounder	Patient encounter
Charlson Comorbidity Index	A measure of patient acuity based on patient age and discharge diagnosis ICD-10^a^ codes assigned after discharge	Confounder	Patient encounter
Discharge to postacute care	Discharge to a setting other than home, including skilled nursing facilities, hospice, and long-term care	Confounder	Patient encounter
Teaching service	Teams that are staffed with a medical student or resident	Confounder	Team
Staffed with an APP^b^	Teams that are staffed with a physician and an APP	Confounder	Team
Starting morning census	The number of patients assigned to a team at 7 AM each morning	Confounder	Team

^a^ICD-10: International Classification of Diseases, 10th Revision.

^b^APP: advanced practice provider.

Surveys were conducted using REDCap (Research Electronic Data Capture)—a secure, web-based application for building and managing web-based surveys and databases [44]—to evaluate the usability of and experience with hospital medicine physicians, APPs, nurses, care management, and other clinical staff during the pilot implementation period. The complete survey results are reported in a study describing the stakeholder engagement and user-centered design approaches that we applied [43].

### Outcomes

Primary outcomes for assessing the effectiveness of this tool were (1) time of day the physician entered the discharge order, (2) time of day the patient left the hospital, and (3) hospital length of stay. Secondary outcomes were (1) proportion of patients for whom a discharge order was entered before 11 AM and (2) proportion of patients discharged before 11 AM, both metrics commonly used to evaluate patient flow. We also queried our data warehouse for the type of patient (inpatient or observation patient), Charlson Comorbidity Index, type of team (physician alone, physician with APP, physician with resident, or physician with APP and resident), proportion of days in the hospital that discharge status was documented for each patient (0%-25%, 25%-50%, 50%-75%, or >75%), and the number of patients assigned to a team at 7 AM (starting morning census).

### Study Size

On the basis of the original planned interrupted time series design, to maximize feasibility against sample size, we allowed for approximately 20 weeks of data collection during each period; that is patients discharged during the preintervention period, patients discharged during the pilot intervention period, and patients discharged during the postintervention period. On the basis of data from 2017, we anticipated an average of approximately 140 discharges per week. However, to account for clustering within providers and teams, the analysis shifted to a mixed modeling approach. Although no post hoc power analysis was conducted, >4000 patients were discharged in each time period.

### Data Analysis

We estimated means and SDs for continuous variables when approximately normally distributed (as assessed by visual inspection of histograms), medians and IQRs when not, and frequencies for categorical variables. Descriptive statistics were computed for patient, clinician, and team characteristics.

Patient-level, clinician-level, and team-level covariates, hypothesized a priori to be associated with the time of discharge order, time of discharge, and hospital length of stay, were included in multivariable analyses. Models for discharge order time, actual discharge time, and hospital length of stay were adjusted for (1) type of patient, (2) Charlson Comorbidity Index, (3) teaching service, (4) staffed with an APP, (5) discharge to postacute care, (6) starting morning census per team, (7) team, and (8) physician (Table 1). The discharge order time and discharge time models were also adjusted for hospital length of stay.

We used linear mixed modeling for the analysis of the time of day the hospitalist physician entered the discharge order into the EHR, the time of day the patient left the hospital, and the hospital length of stay. We converted time to hours elapsed since midnight on a 24-hour clock for modeling. For our binary outcomes, specifically, whether a discharge order was entered before 11 AM and whether a patient was discharged before 11 AM, a generalized linear mixed model with logit link function and binary response distribution was used. The intervention period, that is preimplementation and pilot implementation, was the independent variable of interest. Team and discharging physicians were included as random effects in all models to account for correlation between patients cared for by the same team and the same physician. Given that hospital length of stay is right skewed, this variable was log-transformed to facilitate regression analysis. We reported a relative difference in hospital length of stay by exponentiating the coefficient, subtracting 1, and expressing the result as a percentage [45].

Secondary analyses were performed to determine whether potential effect modification was supported by the data. We hypothesized that the Discharge Today tool would help hospitalist physicians with a high number of patients on their team triage work and enter discharge orders more quickly. To test this hypothesis, we included an interaction term between the team starting morning census and intervention period, allowing for the intervention’s effect to depend on daily patient volume [21,46]. We also hypothesized that the Discharge Today tool might be more effective for teams staffed with an APP, allowing teams to triage and divide work more efficiently [47-49]. To test this hypothesis, we included an interaction term between whether a team was staffed with an APP and intervention period, allowing the intervention’s effect to depend on the presence of an APP.

Patients with missing data on any variables necessary for a specific analysis were excluded from that analysis. All statistical analyses were performed using SAS Enterprise Guide 8.1 (SAS Institute Inc).

## Results

### Use of the Discharge Today Tool

During the preimplementation period—October 1, 2018, to March 4, 2019—4558 patients were discharged from 1 of 18 hospital medicine teams at the University of Colorado Hospital by 57 hospitalist physicians (Table 2). During the pilot implementation period—March 5, 2019, to July 31, 2019—4707 patients were discharged from 1 of 18 teams by 62 hospitalist physicians.

During the implementation period, 84.85% of the patients discharged were assigned a discharge status. The most common barriers identified were medical improvement, placement, subspecialty consults, physical therapy, and social work or care management (Table 3).

**Table 2:** Characteristics of teams, clinicians, and patients by project period.

Characteristics		Preimplementation (N=4558)	Pilot implementation (N=4707)
**Team type, n (%)^a^**			
	With APP^b^	2031 (44.56)	2046 (43.47)
	Without APP	2527 (55.44)	2661 (56.53)
	Teaching	2689 (59.00)	2724 (57.87)
	Nonteaching	1869 (41.00)	1983 (42.13)
Discharges per team, mean (SD)		239.9 (115.8)	247.7 (118.7)
Morning census per team, mean (SD)		10.6 (2.6)	10.7 (2.5)
Unique physicians, n (%)		57 (1.25)	62 (1.32)
Discharges per physician, mean (SD)		72.2 (43.7)	69.1 (46.9)
**Patient type, n (%)**			
	Inpatient	3532 (77.49)	3764 (79.97)
	Observation patient	1004 (22.03)	919 (19.52)
	Missing	22 (0.48)	24 (0.51)
**Discharge disposition, n (%)**			
	Home	3927 (86.16)	4060 (86.25)
	Postacute care setting	557 (12.22)	583 (12.39)
	Other	62 (1.36)	56 (1.19)
	In-hospital death	12 (0.26)	8 (0.17)
Charlson Comorbidity Index, median (IQR)		2 (1-3)	2 (1-3)
**Proportion of days in the hospital a Discharge Today tool status was documented for each patient, n (%)**			
	0%-25% of hospital stay	N/A^c^	401 (8.52)
	26%-50% of hospital stay	N/A	1051 (22.33)
	51%-75% of hospital stay	N/A	798 (16.95)
	>75% of hospital stay	N/A	1253 (26.62)
	Missing	N/A	1204 (25.58)

^a^Teams may fall into more than one category; therefore, the total is >100%.

^b^APP: advanced practice provider.

^c^N/A: not applicable.

**Table 3:** Discharge Today tool use.

Characteristics					Pilot implementation, n (%)
**Discharging hospital medicine physicians (n=56)**					
	Used tool ever			46 (82)	
	Used tool never			10 (18)	
	Used always			16 (29)	
**Patients discharged from a hospital medicine service (n** **=4707)**					
	**Patients ever assigned a discharge status**			3994 (84.85)	
		Ever definite	2087 (52.25)		
		Ever possible	2209 (55.31)		
		Ever tomorrow	N/A^a^		
		Ever in 24-48 hours	1607 (40.24)		
		Ever >48 hours	2771 (69.38)		
		Of the patients ever assigned a discharge status, those with barriers identified	2133 (53.41)		
**Number of barriers identified (n** **=4059)**					
	Medical improvement			1812 (44.64)	
	Placement			532 (13.11)	
	Subspecialty consults			365 (8.99)	
	PT^b^			334 (8.23)	
	Social work or care management			344 (8.48)	
	OT^c^			158 (3.89)	
	RT^d^ or home oxygen			159 (3.92)	
	Transportation			78 (1.92)	
	Test results (laboratory and radiology)			1 (0.02)	
	Follow-up appointment			69 (1.70)	
	IR^e^			66 (1.63)	
	Echo			30 (0.74)	
	Dialysis			36 (0.89)	
	GMT^f^			26 (0.64)	
	Speech			19 (0.47)	
	PICC^g^ line placement			10 (0.25)	
	Pharmacy			13 (0.32)	
	DME^h^			7 (0.17)	
	Wound care			0 (0)	
**Discharge Today tool users** **(n=352)**					
	Registered nurse			71 (20.2)	
	Resident			67 (19.0)	
	Physician			56 (15.9)	
	Physical therapist			31 (8.8)	
	Physician assistant			27 (7.7)	
	Medical student			20 (5.7)	
	Case manager			18 (5.1)	
	Nurse practitioner			15 (4.3)	
	Occupational therapist			12 (3.4)	
	Social worker			9 (2.6)	
	Care coordinator			5 (1.4)	
	Patient resident liaison			4 (1.1)	
	Fellow			3 (0.9)	
	Pharmacist			3 (0.9)	
	Physical therapy student			3 (0.9)	
	Respiratory therapist			2 (0.6)	
	Speech or language pathologist			2 (0.6)	
	Student nurse			1 (0.3)	
	Clinical nurse specialist			1 (0.3)	
	Technician			1 (0.3)	
	Certified nursing assistant			1 (0.3)	

^a^N/A: not applicable.

^b^PT: physical therapy.

^c^OT: occupational therapy.

^d^RT: respiratory therapy.

^e^IR: interventional radiology.

^f^GMT: glucose management team

^g^PICC: peripherally inserted central catheter.

^h^DME: durable medical equipment.

Of the 56 hospitalists who discharged a patient during the pilot implementation period, 46 (82%) used the tool for patients assigned to their teams. During the pilot implementation period, 352 users, including physicians, APPs, residents and medical students, nurses, physical and occupational therapists, care managers and social workers, and pharmacists, added the tool to their patient worklists. Of these users, 86% (48/56) of hospitalist physicians and 88% (29/33) of hospitalist APPs added the tool to their EHR patient lists. Physicians, APPs, residents, and medical students added the primary column in which they entered a discharge readiness status daily, and other clinical staff, including nurses, physical and occupational therapists, care managers and social workers, and pharmacists, added the read-only columns where the discharge readiness status entered by providers can be viewed. In addition, in some cases, the tool was added to shared patient worklists, which meant that >352 clinical staff were using the tool.

Hospital medicine physicians, APPs, nurses, care management, and other clinical staff reported in a survey conducted during the pilot implementation period that the tool did not adversely affect their workflow (21/24, 88% of the providers, and 34/34, 100% of clinical staff) and was helpful for managing the patient discharge process (32/34, 94% of clinical staff).

### Effectiveness of the Discharge Today Tool

In both unadjusted effectiveness analysis and after adjusting for prespecified confounders, we did not find a significant reduction in the time of day the discharge order was entered into the EHR by the discharging physician during the pilot implementation period compared with the preimplementation period (Table 4).

**Figure 2:**
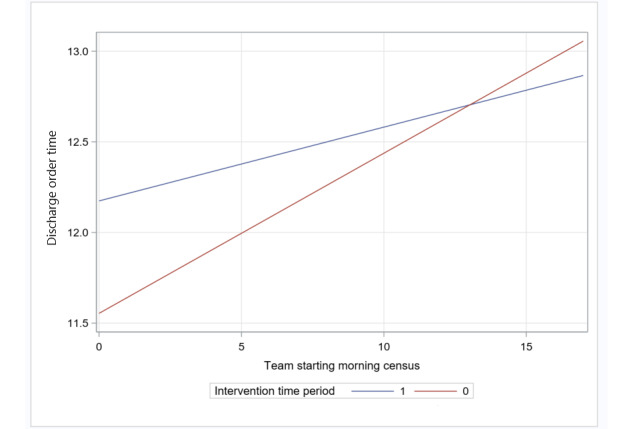
Discharge order time: interaction between team starting morning census and intervention period (preimplementation vs pilot implementation).

In the secondary analyses for effect modification, we observed an interaction effect between intervention period and starting morning census (*P=*.07; Figure 2).

The time of day the discharge order was entered into the EHR by the discharging physician varied according to the number of patients assigned to a team at 7 AM each morning in the pilot implementation period compared with the preimplementation period.

Specifically, the time of day the discharge order was entered into the EHR by the discharging physician decreased by an additional 2.9 minutes per patient for every 1-patient increase in morning census during the pilot implementation period compared with each 1-patient increase in morning census during the preimplementation period. However, we did not find any evidence of effect modification for the intervention by the presence of an APP (Table 4).

**Figure 3:**
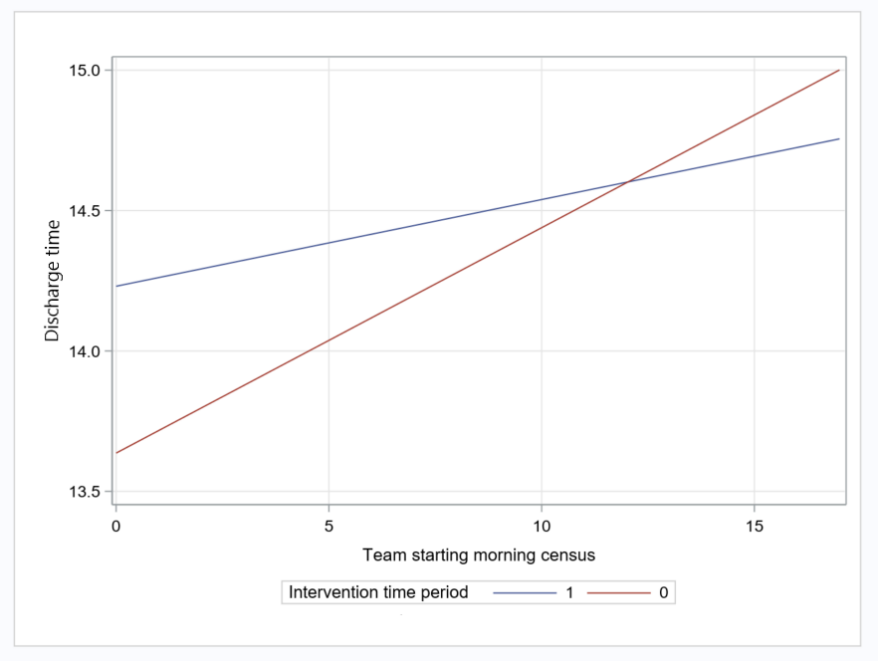
Discharge time: interaction between team starting morning census and intervention period (preimplementation vs pilot implementation).

Although in unadjusted and adjusted analyses the time of day the patients left the hospital for the pilot implementation period compared with the preimplementation period did not change significantly, we found, in secondary analyses conducted to investigate effect modification, that the average time of day the patients left the hospital decreased for every 1-patient increase in morning census for a given team during the preimplementation period compared with the pilot implementation period by 3.0 minutes (*P=*.07; Figure 3).

**Table 4:** Discharge Today tool effectiveness modeling by project period.

Characteristics			Discharge order time, mean (SD)^a^		Discharge time, mean (SD)^a^		Length of stay in hours, median (IQR)^b^		Discharge order before 11 AM^c^, n (%)		Discharge before 11 AM^c^, n (%)
Preimplementation (N=4114)			12:40 (2:38)		14:41 (2:46)		75 (47-138)		1125 (27.35)		382 (9.29)
Pilot implementation (N=4285)			12:45 (2:33)		14:44 (2:43)		76 (46-139)		1103 (25.74)		367 (8.56)
**Unadjusted results**											
	95% CI	5.1 (–3.8 to 14.1)		2.1 (–6.6 to 10.9)		1.9 (–6 to 2.3)		0.94 (0.83 to 1.1)		0.91 (0.77 to 1.1)	
	*P* value	.26		.63		.37		.35		.26	
**Adjusted results**											
	95% CI	6.8 (–2.2 to 15.8)		4.0 (–5.2 to 13.2)		3.7 (–7.4 to 0.1)		0.92 (0.81 to 1.0)		0.90 (0.76 to 1.1)	
	*P* value	.14		.39		.06		.19		.22	
**Starting morning census**											
	95% CI	–2.9 (–5.9 to 0.2)		–3.0 (–6.2 to –0.2)		0.3 (–1.7 to 1.1)		1.0 (0.97 to 1.1)		1.1 (0.98 to 1.1)	
	*P* value	.07		.07		.66		.66		.16	
**Staffed with an APP^d^**											
	95% CI	–9.2 (–27.1 to 8.6)		–19.1 (–37 to –0.9)		4.7 (–11.9 to 3.1)		1.1 (0.84 to 1.4)		1.4 (0.99 to 2.0)	
	*P* value	.31		.04		.23		.53		.06	

^a^Mean difference (in minutes) calculated.

^b^Mean percentage decrease calculated.

^c^Odds ratio calculated.

^d^APP: advanced practice provider.

The time of day the patient left the hospital after being discharged varied according to the number of patients assigned to a team at 7 AM each morning in the pilot implementation period compared with the preimplementation period.

**Figure 4:**
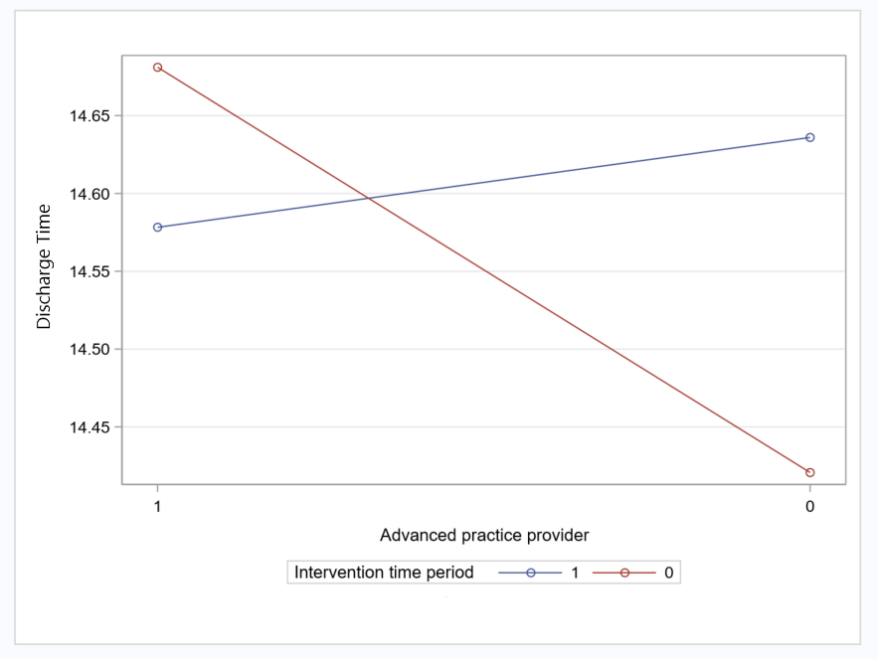
Discharge time: interaction between team staffed with an advanced practice provider and intervention period (preimplementation vs pilot implementation).

In addition, the average time of day the patients left the hospital decreased for teams staffed with an APP during the preimplementation period compared with the pilot implementation period by 19.1 minutes (*P=*.04; Table 4; Figure 4).

The time of day the patient left the hospital after being discharged varied according to whether a team was staffed with an advanced practice provider in the pilot implementation period compared with the preimplementation period.

In the unadjusted analysis, hospital length of stay did not change significantly. After adjusting for prespecified confounders, we observed a trend toward reduction in hospital length of stay for the pilot implementation period compared with the preimplementation period (decrease of 3.7%; *P=*.06). We did not observe significant changes in the length of stay from preimplementation to pilot implementation under different conditions, such as high-census days or presence of an APP on a patient team; that is, no significant interactions between the intervention period and these variables were detected (Table 4).

Neither of the secondary outcomes—proportion of patients for whom a discharge order was entered before 11 AM and proportion of patients discharged before 11 AM—was found to significantly improve after introduction of the Discharge Today tool in unadjusted analysis, after adjusting for prespecified covariates, or under different conditions (Table 4).

**Figure 5:**
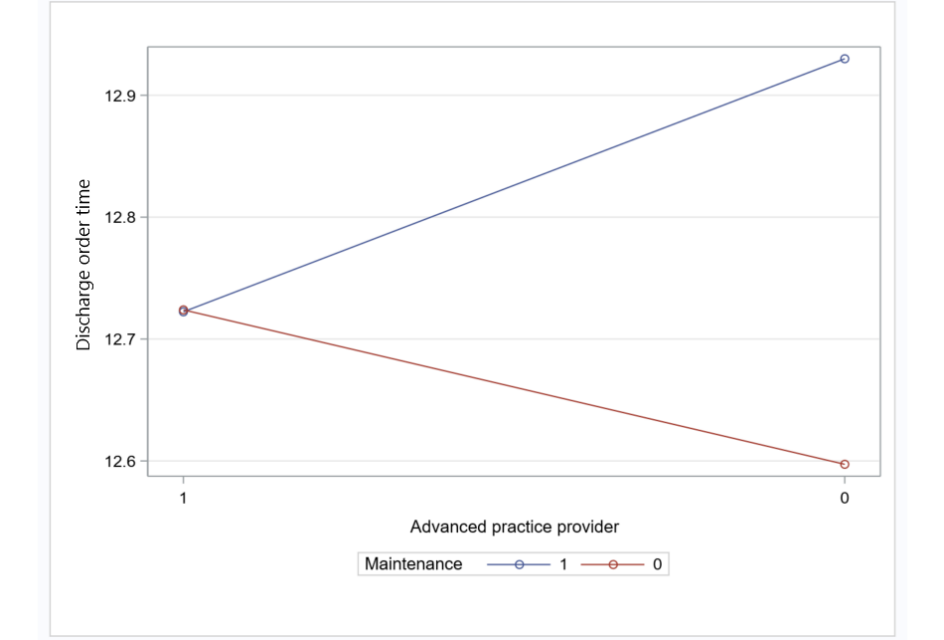
Discharge order time: interaction between team staffed with an advanced practice provider and intervention period (pilot implementation vs postimplementation).

To test whether the effects of this tool persisted in a maintenance period during which stakeholder engagement efforts were curtailed, we compared the outcomes of the pilot implementation period with those of the postimplementation period using mixed effects models (Table 5). Adjusting for prespecified covariates, we observed a significant reduction in the time of day the discharge order was entered into the EHR for teams staffed with an APP during the postimplementation period compared with teams staffed with an APP during the pilot implementation period (an average decrease of 20.1 minutes per patient (95% CI –36.1 minutes to –4.0 minutes; *P=*.01; Figure 5).

The time of day the discharge order was entered into the EHR by the discharging physician varied according to whether a team was staffed with an advanced practice provider in the postimplementation (maintenance) period compared with the pilot implementation period.

However, no other outcomes improved significantly from the pilot implementation period to the postimplementation period.

**Table 5:** Discharge Today tool effectiveness modeling comparing pilot implementation and postimplementation periods.

Characteristics			Discharge order time^a^, mean (SD)		Discharge time^a^, mean (SD)		Length of stay in hours^b^, median (IQR)		Discharge order before 11 AM^c^, n (%)		Discharge before 11 AM^c^, n (%)
Pilot implementation (N=4285)			12:45 (2:33)		14:44 (2:43)		76 (46-139)		1103 (25.74)		367 (8.56)
Postimplementation (N=4255)			12:56 (2:29)		14:53 (2:38)		79 (47-142)		924 (21.72)		327 (7.69)
**Unadjusted results**											
	95% CI	11 (3.1 to 18.9)		9.8 (2.0 to 17.6)		4.9 (0.1 to 9.9)		0.80 (0.70 to 0.91)		0.89 (0.74 to 1.1)	
	*P* value	.01		.01		.04		.001		.2	
**Adjusted results**											
	95% CI	11 (2.8 to 19.1)		10.1 (2.0 to 18.1)		5.3 (1.1 to 9.7)		0.81 (0.71 to 0.92)		0.87 (0.72 to 1.1)	
	*P* value	.01		.01		.01		.002		.14	
**Starting morning census**											
	95% CI	–0.1 (–3.0 to 2.8)		0.1 (–2.9 to 3.1)		1.9 (0.5 to 3.4)		0.99 (0.95 to 1.0)		0.98 (0.92 to 1.1)	
	*P* value	.96		.95		.01		.75		.63	
**Staffed with an APP^d^**											
	95% CI	–20.1 (–36.1 to –4.0)		–11.7 (–27.6 to 4.3)		1.9 (–6.2 to 10.7)		1.3 (1.0, 1.7)		1.25 (0.86 to 1.8)	
	*P* value	.01		.15		.66		.05		.25	

^a^Mean difference (in minutes) calculated.

^b^Mean percentage decrease calculated.

^c^Odds ratio calculated.

^d^APP: advanced practice provider.

## Discussion

### Principal Findings

The important findings of this work are as follows:

1. There was considerable uptake and use of the Discharge Today tool for the duration of the study period, with most clinicians adding it to their patient lists in the EHR and providing discharge updates for most patients.
2. The surveyed providers and clinical staff reported that the tool was efficient to use, did not adversely affect their workflow, and was helpful for patient discharge management.
3. After adding teams staffed with an APP as an effect modifier, for teams that included an APP, there was a significant reduction in the time of day the patient left the hospital beyond the reduction seen for teams without an APP.

Other studies have described similar tools, such as the Red, Yellow, or Green Discharge tool [50] and the Kanban web-based application [51]. However, these tools were not integrated into the patient worklist, an EHR workspace that is commonly used across clinical staff, including physicians, APPs, residents, medical personnel, nurses, physical and occupational therapists, care managers and social workers, and pharmacists, thus enhancing the real time, multidisciplinary communication about discharge readiness. Recently, a similar tool was described in the pediatric setting, which was associated with an increase in the proportion of patients discharged before noon [52]. However, unlike our Discharge Today tool, this tool did not allow providers to document any tasks or clinical care required before the patient could be discharged. In addition, our Discharge Today tool allowed providers to note what time of day (before 11 AM, before 2 PM, or after 2 PM) a patient might be discharged.

Previous work has shown that hospital census and census on teams can affect the overall flow of hospitals [21,46]. In this pilot study, although the primary outcomes evaluated were nonsignificant in analysis, interactions between the number of patients assigned to a team in the morning or teams staffed with an APP and the intervention suggest that there may be effect modification at work such that the intervention is effective in certain subgroups or under certain conditions. After including starting morning census per team as an effect modifier, although nonsignificant, there was a reduction in the time of day the discharge order was entered into the EHR by the discharging physician and in the time of day the patient left the hospital. In addition, when teams were staffed with an APP, the use of this tool was associated with significantly earlier discharges beyond that seen for teams without an APP. Research has shown that discharging patients just 1 hour earlier alleviates hospital crowding and reduces access blocking [23,24]. Although we were not able to achieve the goal of discharging patients an hour earlier on average, the incremental gains from multiple solutions implemented across many patients may be additive to moving discharge times and could result in improvements in patient flow and hospital capacity.

Finally, during the maintenance period, when teams were staffed with an APP, discharge orders were entered significantly earlier by the discharging providers. Our APPs were early adopters of the tool and continue to be heavy users, which may have produced the observed improvements. We believe that these findings highlight the importance of APPs in the success of discharge initiatives. Although other studies have suggested that a multidisciplinary approach will improve the early discharge of patients [22,53,54], our study specifically investigates the effects of APP involvement. Our study suggests that APPs may be vital partners in work undertaken to improve the discharge process in an adult medicine population. A pilot study of a multidisciplinary team led by an APP and staffed by a pharmacist and nurse demonstrated a significant improvement in discharge times for patients seen by this team [55]. Similarly, previous research has shown that most providers do not prioritize discharges first as they are tending to other patients [21]; thus, using a team approach to patient care may be advantageous when working to improve throughput metrics.

Our results suggest that some effects of the tool continued even after robust stakeholder engagement efforts were reduced to periodic reminders. We observed a significant reduction in the mean time of day the physician entered the discharge order when a team was staffed with an APP over the reduction observed when a team was not staffed with an APP during the postimplementation period compared with the pilot implementation period. During this time, the hospital medicine triagist, an APP-staffed position, started using the tool for bed management, suggesting that APP use may have become more deliberate. Sustained improvement after demonstrating the effectiveness of an intervention is not often evaluated, likely because of constraints of time and available budget [56]; however, without consideration of the relevant contextual factors, evaluating whether an intervention has resulted in sustainable improvement may prove elusive [57].

Finally, our tool had high adoption and use rates, with relatively minimal incentives to do so. There were several features of our project that helped to improve adoption and use. Our stakeholder engagement process—both preimplementation and during the pilot implementation period—was robust, resulting in a product that was developed for and by frontline staff members and clinicians. In addition, the Discharge Today tool was integrated into the current workflow (ie, EHR worklists) and color coded, which serves as a visual prompt for both clinicians and frontline staff to use.

Given that this was a pilot study of this tool aiming to evaluate the user-centered design approach taken, adoption of the tool, and effectiveness in a sample of providers delivering care to hospitalized patients, the tool had not been fully scaled up across hospital settings, thus potentially limiting the effectiveness. Although our tool had high adoption rates with our target populations, it was challenging to fully implement it across all care teams across an entire hospital, and thus it took some time to scale. Since this pilot, an initiative to use the existing EHR applications to better support patient flow has been launched. As an aspect of this work, the Discharge Today tool has been integrated with other EHR functionalities to capture patients’ progress toward discharge, and any roadblocks to discharge were implemented. We suspect that as adoption continues and additional features are added, adoption will further scale, and perhaps larger effects on the desired outcomes could be seen. EHR tools intended to change clinician behavior require continuous iterative optimization and evaluation to realize their full potential.

Our study has a number of strengths. First, we describe a novel tool to communicate discharge readiness in real time to key stakeholders. Second, we had remarkable engagement by our clinicians and frontline staff members, with high use rates and overall positive feedback. Third, although our study was conducted at a single center, our sample included >4000 patients, almost 60 physicians, >40 APPs, almost 90 residents and medical students, and >160 frontline staff members during the pilot implementation period. Fourth, we have accounted in statistical modeling for the contextual factors that we hypothesized a priori could influence the effectiveness of the tool by including effect modifiers for the number of patients assigned to a team in the morning and a team staffed with an APP.

Our study also has several limitations. First, it was performed at a single university-affiliated academic hospital and was a quality improvement initiative using a pre-post study design; therefore, the results might not be generalizable to other types of institutions or other patient populations. Second, throughout our study period, we continued to optimize the tool, and thus the full effect of the tool may not have been realized at the end of the pilot implementation period. Third, for this analysis, we assessed both the discharge order time and the discharge time; however, we did not evaluate the circumstances around that gap (ie, when the patient was actually ready for discharge and any reasons for delays between the time the order was entered and the time the patient could leave). Future analyses would benefit from assessing whether the use of the Discharge Today tool closes the gap between when the discharge order is entered and when the patient is actually discharged. Fourth, there are most likely unknown confounders at work that we did not identify or include as adjustment factors. Fifth, although we did ask the providers to update the tool first thing in the morning and throughout the day as patient statuses changed, we did not ask that they otherwise change their workflow. Before, during, and after this study, there have been consistent institutional efforts asking providers to prioritize discharges first. It is possible that by asking providers to update the status, that alone could have resulted in improved discharge times regardless of the tool used; however, even with discharge-before-noon initiatives implemented at most places, <10% of hospitalists typically round on discharges first [21]. On the basis of previous literature and mixed successes around early-discharge initiatives, we believe that a multipronged approach is likely needed, including ensuring reasonable workloads, optimizing care team models, and improving communication processes [4,35,50,58]. This tool offers a potential component that is minimally intrusive and communicates across disciplines.

Finally, we were unable to account for other initiatives (eg, huddles held throughout the day to discuss patients who may be able to be discharged) intended to improve discharge times and lengths of stay that were taking place concurrently with our Discharge Today tool implementation.

### Conclusions

We have described a unique, EHR-based approach to improving communication around discharge in real time with all care team members, regardless of their physical location in the hospital, that improves discharge times and lengths of stay. The Discharge Today tool allows for real time documentation and sharing of discharge statuses, and our results suggest that the tool may be useful for improving discharge times and lengths of stay, particularly if a team is staffed with an APP or possibly in higher-census situations.

## Abbreviations

APP: advanced practice provider
EHR: electronic health record
REDCap: Research Electronic Data Capture
